# Anti-NMDA receptor encephalitis following Murray Valley encephalitis: a case report

**DOI:** 10.1128/asmcr.00025-24

**Published:** 2025-02-12

**Authors:** Arvind Yerramilli, Prashanth Ramachandran, John Burston, Annaleise R. Howard-Jones, Chuan Kok Lim, Jen Kok, Leigh Fitzpatrick, Peter Hingston, Justin Jackson

**Affiliations:** 1 Department of Medicine, Albury-Wodonga Health, Albury, New South Wales, Australia; 2 Department of Infectious Diseases at the Peter Doherty Institute for Infection and Immunity, Melbourne, Victoria, Australia; 3 Department of Neurology, The Royal Melbourne Hospital, Parkville, Victoria, Australia; 4 Department of Neurology, St. Vincent’s Hospital, Fitzroy, Victoria, Australia; 5 Charles Sturt University School of Rural Medicine, Albury, New South Wales, Australia; 6 Department of Infectious Diseases and Microbiology, The Children’s Hospital at Westmead, Westmead, New South Wales, Australia; 7 Sydney Institute for Infectious Diseases, The University of Sydney 4334 , Sydney, New South Wales, Australia; 8 Victorian Infectious Diseases Reference Laboratory, The Royal Melbourne Hospital at the Peter Doherty Institute for Infection and Immunity, Melbourne, Victoria, Australia; 9 Center for Infectious Diseases and Microbiology Laboratory Services, NSW Health Pathology-Institute of Clinical Pathology and Medical Research, Center for Infectious Diseases-Public Health, Westmead Hospital, Westmead, New South Wales, Australia; 10 Faculty of Medicine, University of New South Wales (UNSW) Rural Clinical School, Albury, New South Wales, Australia; Vanderbilt University Medical Center, Nashville, Tennessee, USA

**Keywords:** Murray Valley encephalitis, autoimmune encephalitis, anti-NMDA receptor encephalitis, post-viral encephalitis, flavivirus, Australia

## Abstract

**Background:**

Anti-N-methyl-D-aspartate (NMDA) receptor encephalitis is an autoimmune condition that is increasingly being recognized as a complication of central nervous system infections. While association with herpes simplex virus encephalitis is well established, the condition has also been reported to occur following Japanese encephalitis virus infection and yellow fever vaccination.

**Case Summary:**

Here, we report a case of anti-NMDA receptor encephalitis following infection with Murray Valley encephalitis virus (MVEV), another virus of the family Flaviviridae. Following initial recovery, and 34 days after the onset of illness, our patient developed autonomic instability, orofacial dyskinesia, and decreased level of consciousness. Diagnosis was established with the detection of anti-NMDA receptor antibodies in cerebrospinal fluid. Immunosuppressive therapy with rituximab and corticosteroids resulted in a favorable outcome.

**Conclusion:**

This case report raises awareness of post-viral autoimmune encephalitis which should be considered in patients with a biphasic pattern of illness following viral encephalitis or a compatible clinical syndrome. Given the initial diagnosis of Murray Valley encephalitis, clinicians should be alert to the possibility of MVEV as an infectious trigger associated with the development of anti-NMDA receptor encephalitis. We also highlight the challenges inherent in the diagnosis of flavivirus infections, which may require a combination of clinical, epidemiological, and laboratory evidence.

## INTRODUCTION

Anti-N-methyl-D-aspartate receptor (anti-NMDAR) encephalitis is an autoimmune condition originally described in association with ovarian teratomas and subsequently recognized to follow central nervous system (CNS) infection with herpes simplex virus (HSV) (1–3). An association has also been reported with Flaviviridae, including yellow fever vaccination and Japanese encephalitis virus (JEV) infection (4–6). Here we introduce a case of anti-NMDAR encephalitis that occurred following CNS infection with Murray Valley encephalitis virus (MVEV) and highlight the importance of recognition and appropriate management of this condition.

## CASE PRESENTATION

A 21-year-old male presented in March 2023 to a regional health service on the border of Victoria and New South Wales, Australia, with a 5-day history of headache and fever that progressed to ataxia and confusion. His medical history was significant for obesity, migraine, generalized anxiety disorder, and level 1 autism spectrum disorder. Seven days prior to the presentation, he had been on a camping trip along the Murray River. The patient worked at a local piggery and had not been vaccinated against JEV. Examination revealed a temperature of 40.1°C with sinus tachycardia of 140 beats/minute. There was evidence of bilateral upper and lower limb ataxia on neurological assessment.

Initial investigations including full blood examination revealed a leukocytosis of 16.8 × 10^9^ cells/L (reference range 4.0–11.0) with neutrophilia of 13.4 × 10^9^ cells/L (reference range 2.0–8.0) and a C-reactive protein of 16 mg/L (reference range 0–10). Computed tomography of the brain without contrast was unremarkable. Cerebrospinal fluid (CSF) analysis also collected prior to antimicrobials revealed a normal glucose of 3.4 mmol/L (reference range 2.0–3.9) but raised protein of 1.22 g/L (reference range 0.15–0.45). There was a predominant lymphocytic pleocytosis with white cell count of 303 × 10^6^ cells/L (12 × 10^6^ cells/L polymorphs and 291 × 10^6^ cells/L mononuclear cells). No organisms were seen on Gram stain, and there was no growth on cultures at 48 hours of incubation. Targeted reverse-transcriptase PCR for enteroviruses as well as HSV and varicella zoster virus was negative, as was the BioFire FilmArray Meningitis/Encephalitis Panel (BioMérieux). Anti-NMDAR antibodies were not detected on CSF (Euroimmun Anti-Glutamate Receptor [Type NMDA] Indirect Immunofluorescence). Cryptococcal antigen was not detected in serum and CSF. Combined HIV antigen and antibody testing on serum was negative.

**Fig 1:**
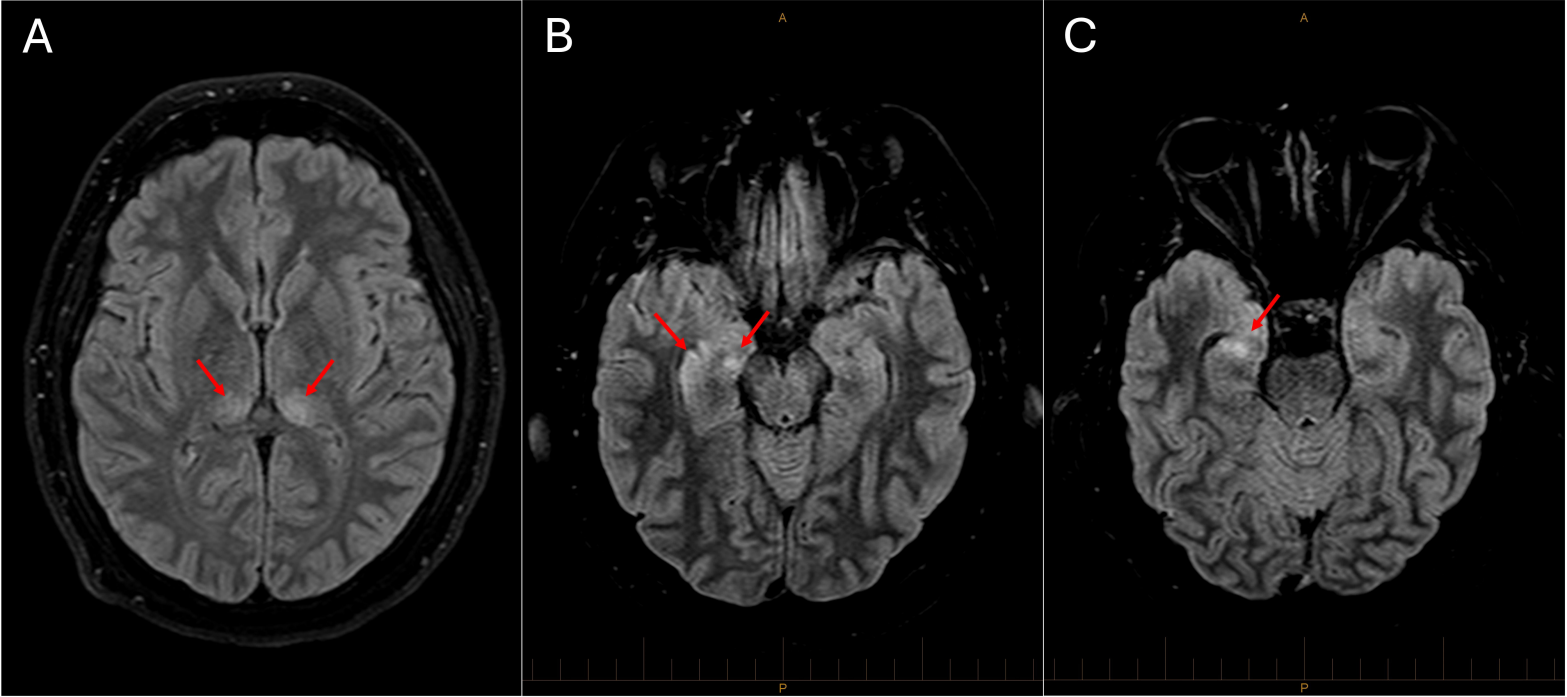
MRI of the brain showing signal hyperintensity in the bilateral posteromedial thalami (panel A) and right mesial temporal lobe structures including the hippocampus and parahippocampal gyrus (panels B and C).

The patient was commenced on empiric meningoencephalitis antimicrobial treatment with ceftriaxone 2 g intravenously (IV) every 12 hours, benzylpenicillin 2.4 g IV every 4 hours, and acyclovir 10 mg/kg IV every 8 hours along with dexamethasone 10 mg IV every 6 hours. A decreased conscious state with airway obstruction, alveolar de-recruitment, and hypoxic respiratory failure necessitated intubation and mechanical ventilation. Magnetic resonance imaging (MRI) of the brain showed a hyperintense signal within the right mesial temporal lobe and bilateral posteromedial thalami (Fig. 1).

CSF and blood samples were tested across two reference laboratories for JEV, MVEV and West Nile Virus/Kunjin subtype (KUNV) using nucleic acid and serological assays and algorithms as previously described (7, 8). Testing of both serum and CSF samples collected on presentation (day 0) for all flaviviruses was negative in reference laboratory 1 (Table 1). Repeat testing of serum collected on day 10 following presentation and tested in parallel with the day 0 serum demonstrated seroconversion of JEV-specific IgM and IgG as well as MVEV-specific total antibodies. There was insufficient sample to titrate the MVEV antibodies. Using a different assay in reference laboratory 2, MVEV- but not JEV-specific total antibodies were detected in the same sample collected on day 10 (9). MVEV-specific IgM was detected in a further sample collected on day 20; however, neither JEV-specific total antibody nor KUNV-specific IgM was detected, providing laboratory suggestive evidence for MVEV infection (10). This sample demonstrated a high titer to MVEV-specific total antibody at 1:640. KUNV-specific but not JEV-specific total antibody was also detectable in this sample albeit at low static titers, consistent with either prior infection or cross reactivity. Serial testing of day 20 and day 30 samples in reference laboratory 1 showed largely static results, while a reduction in titers for MVEV at day 30 was seen in reference laboratory 2.

**TABLE 1:** Serial flavivirus testing results^*d*^

Method	Specimen type	Test	Day 0	Day 10	Day 20	Day 30	Day 48
Molecular	CSF	Flavivirus RT-PCR	Not detected				Not detected
Antibody	CSF	JEV IgM IFA	Not detected				Not detected
		JEV IgG IFA	Not detected				Not detected
		MVEV total Ab ELISA	Not detected				Not detected
		KUNV total Ab ELISA	Not detected				Not detected
Serological platform 1	Serum	JEV IgM IFA	Not detected	Detected^*a*^	80	40	
		JEV IgG IFA	Not detected	160	160	320	
		MVEV total Ab DEB-ELISA	Not detected	Detected^*a*^	Detected^*a*^	Detected^*a*^	
		KUNV total Ab DEB-ELISA	Not detected	Not detected	Not detected	Not detected	
Serological platform 2	Serum	JEV total Ab DEB-ELISA		Not detected	Not detected	Not detected	
		JEV IgM IFA		¶^*b*^	¶^*b*^	¶^*b*^	
		MVEV total Ab DEB-ELISA / IFA		Detected^*a*^	640	160	
		MVEV IgM IFA		*^c^	80	40	
		KUNV total Ab DEB-ELISA / IFA		*^c^	40	20	
		KUNV IgM IFA		*^c^	Not detected	Not detected	

^*a*^ Insufficient sample to perform titer value.

^*b*^ ¶, not tested in the context of negative JEV total antibody, as per protocol.

^*c*^ *, insufficient sample for further testing.

^*d*^ RT-PCR = reverse transcriptase PCR; IFA = indirect immunofluorescence assay; ELISA = enzyme-linked immunosorbent assay; DEB = defined epitope blocking assay.

The patient gradually improved with continued supportive therapy. His cognitive state returned to normal, his fevers settled, and he was able to obey complex commands. However, 34 days from the illness onset, fever returned, and the patient became tachycardic. Four days later, he developed abdominal distension due to bowel ileus requiring the insertion of a nasogastric tube. By 40 days from the illness onset, he had lost purposeful communication and developed further autonomic instability along with widespread dyskinetic movements predominantly involving the face and hands. Intravenous methylprednisolone 1 g daily for 5 days was commenced while further investigations were undertaken, without clinical improvement.

**Fig 2:**
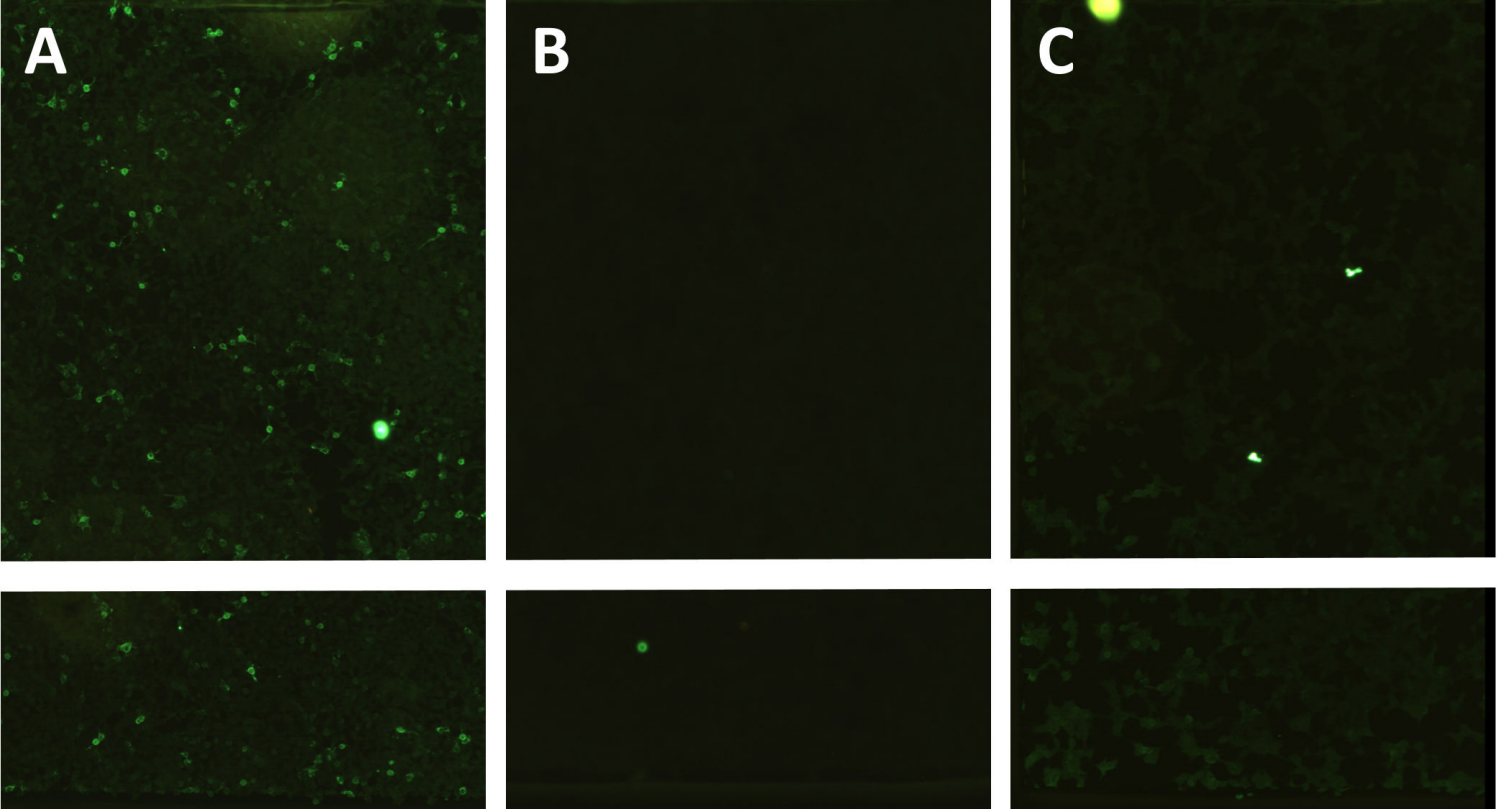
Indirect immunofluorescence assays of anti-NMDA receptor antibodies showing a positive result on CSF neat (A), negative result on CSF at dilution 1:100 (B), and positive result on serum at dilution 1:10 (C).

A repeat MRI of the brain demonstrated stable distribution and extent of the previously documented findings. An electroencephalogram excluded status epilepticus as a cause for his neurological deterioration. A repeat lumbar puncture was performed on day 48 of illness, and CSF analysis was again abnormal with 11 × 10^6^ /L white cells (100% mononuclear cells), protein of 1.65 g/L, and mildly elevated glucose (4.1 mmol/L) with a matched capillary blood glucose of 6.2 mmol/L. No organisms were seen on Gram stain, and no pathogens were isolated in the culture. Flavivirus PCR and antibody testing were again negative (Table 1). Oligoclonal immunoglobulin G bands were detected. Anti-NMDA receptor antibodies were detected in both serum and the CSF from the second lumbar puncture confirming a diagnosis of anti-NMDAR encephalitis (Euroimmun Anti-Glutamate Receptor [Type NMDA] Indirect Immunofluorescence). Serum antibodies were only tested at a dilution of 1:10 and were positive, while CSF antibodies were strongly positive neat and negative at a dilution of 1:100 (Fig. 2).

Intravenous immunoglobulin product was administered at a dose of 100 g daily (1 mg/kg) for 5 days, also without clinical improvement. Our patient proceeded to rituximab, receiving two doses 2 weeks apart of 1,000 mg each, combined with a weaning course of oral corticosteroids. Following rituximab, he made a steady neurological recovery with improvement in his autonomic and cognitive symptoms. The patient was discharged from the hospital 6 months after the presentation. On follow-up 11 months later, he was no longer taking oral steroids and was mobilizing independently. He had ongoing mild short-term memory loss, mild bilateral parkinsonism with cogwheel rigidity, and decrementing bradykinesia, although without significant functional impairment.

## DISCUSSION

JEV, MVEV, and KUNV are Flaviviridae which are carried by mosquito vectors with waterbirds serving as primary reservoirs. In Australia, outbreaks have been associated with La Niña weather patterns, which lead to above-average rainfall and widespread flooding events (11). Expanded inland water bodies are hypothesized to result in the migration of viremic waterbirds, increased mosquito vector prevalence, and the consequent epizootic spread of flaviviruses (11). Historically, the predominant cause of encephalitis in Australia has been MVEV (12), with KUNV only rarely reported (13). From July 2020 through to March 2023, Australia had 3 consecutive years of La Niña weather patterns. It was during the second year of this event that Australia recorded the first-ever outbreak of JEV, consisting of 45 human cases (35 confirmed and 10 probable) (14), with the sentinel cluster of cases recognized in patients presenting to our facility in February 2022 (15). No further cases of JEV have been reported since December 2022 (14). On 16 January 2023, during the third La Niña year, an outbreak of MVEV was declared, with 27 cases identified nationwide between January and July 2023 (16). Our case was presented during this latter outbreak.

In Australia, JEV, MVEV, and KUNV should be considered as differential causes of encephalitis. Laboratory confirmation of infection requires either RNA detection or isolation of the virus, or demonstration of seroconversion or a significant rise in antibody titers. Based on the results of serology testing, our patient fulfilled the case definition for a probable case of MVEV which was also consistent with the local contemporaneous epidemiology (10). All diagnostic assays have varying analytical specificities and sensitivities, and this case highlights that inherent variability and the potential for diagnostic confusion. Flavivirus serology is particularly prone to this phenomenon due to shared epitopes and anamnestic immune responses, and, hence, the potential for cross-reacting antibodies (17). Serial testing and/or confirmatory testing using different assays in different laboratories may be required to achieve a definitive diagnosis. Compared to serology, the detection of nucleic acid in multiple sample types (such as CSF, blood, urine, and/or brain tissue) greatly enhances specificity and improves diagnostic yield. For this case, clinical, epidemiological, and serological evidence supported a diagnosis of MVEV as the cause of our patient’s initial illness.

Anti-NMDAR encephalitis was first described in 2007 in association with ovarian teratoma and the detection of autoantibodies against the NMDA receptor (1, 2). Subsequently, viral encephalitis has also been recognized to trigger anti-NMDAR encephalitis and other autoimmune encephalitides. In 2014, Armangue and colleagues demonstrated that post-viral autoimmune encephalitis targets the NMDA and other neurotransmitter receptors with demonstrable antibodies in serum and CSF (3). Since 2014, other infectious triggers of autoimmune encephalitis have also been recognized. Anti-NMDAR encephalitis has been described following yellow fever vaccination, with four cases published to date (4). In 2017, Ma et al. reported three pediatric cases of JEV-induced anti-NMDAR encephalitis developing 3–4 weeks following the first presentation, all with confirmation of JEV IgM antibodies in serum and CSF (5). All three cases had detectable anti-NMDAR antibodies in CSF, and one also had detectable antibodies in serum. A subsequent prospective observational study including 65 patients with CSF-confirmed Japanese encephalitis (JE) demonstrated the development of autoimmune encephalitis in five (7.9%) with the detection of anti-NMDAR antibodies in the CSF of three of these cases (6). From a mechanistic standpoint, the NMDAR signal transduction pathway appears to be related to JE neuronal cell death pathogenesis. Chen and colleagues demonstrated that activation of NMDAR after JEV infection leads to increased neuronal damage both *in vitro* via mouse neurons and glial cultures, as well as *in vivo* mouse models of infection (18). These findings suggest that the NMDAR could play an important role in flavivirus pathogenicity and may be aberrantly triggered by such infections with the potential for resultant immune dysregulation.

Our case demonstrates a potential association between MVEV, another member of the flavivirus genus, and anti-NMDAR encephalitis. It is unclear if this is a bystander effect or causation as intrathecal antibody response post-flavivirus infection is poorly characterized. Nevertheless, like other descriptions of post-viral autoimmune encephalitis, our patient followed a biphasic pattern of illness. The median time for the development of autoimmune encephalitis reported by Armangue et al. was 32 days, with a range of 7–61 days (19). This timeframe is consistent with our patient’s illness, with symptom onset 34 days from his initial presentation. Anti-NMDAR encephalitis also has characteristic clinical findings and is frequently recognizable based on the clinical syndrome (2, 20, 21). Psychiatric manifestations tend to be prominent, as does disordered sleep. These changes may be followed by speech dysfunction, dyskinesias, autonomic instability, and a decrease in the level of consciousness. Seizures may occur at any time. Our patient demonstrated autonomic instability associated with orofacial and hand dyskinesia, as well as a decreased conscious state. CSF IgG autoantibodies against the GluN1 subunit of the NMDA receptor are highly specific, as seen in our patient.

Regarding post-JEV anti-NMDAR encephalitis, response to immunotherapy was reported by Ma et al.; however, treatment details were not provided, the sample size was small (*n* = 5), and follow-up was short (range of 32–41 days post-symptom relapse) (6). In the series of autoimmune encephalitis following HSV reported by Armangue et al., treatment regimens included methylprednisolone, plasma exchange, and rituximab (19). Infants had a worse prognosis with more residual comorbidity, whereas the predominant deficit in adults was anterograde amnesia. While dramatically improved, at 10 months, our patient also had residual neurological deficits including memory impairment and parkinsonism, although the latter likely related to the initial flavivirus infection and basal ganglia abnormalities.

In conclusion, this case report raises awareness of post-viral autoimmune encephalitis and adds MVEV to the list of infectious and non-infectious triggers associated with the development of anti-NMDAR encephalitis. Clinicians should be alert to the possibility of autoimmune encephalitis in patients with a biphasic pattern of illness following viral encephalitis or a compatible clinical syndrome. Recognition is important, as prompt treatment with immunotherapy is key to minimizing mortality and long-term neurological sequelae.
